# Association of immune cell subsets with cardiac mechanics in the Multi-Ethnic Study of Atherosclerosis

**DOI:** 10.1172/jci.insight.149193

**Published:** 2021-07-08

**Authors:** Arjun Sinha, Adovich S. Rivera, Margaret F. Doyle, Colleen Sitlani, Alison Fohner, Sally A. Huber, Nels C. Olson, Joao A.C. Lima, Joseph A. Delaney, Matthew J. Feinstein, Sanjiv J. Shah, Russel P. Tracy, Bruce M. Psaty

**Affiliations:** 1 Department of Medicine and; 2 Department of Preventive Medicine, Feinberg School of Medicine, Northwestern University, Chicago, Illinois, USA.; 3 Department of Pathology and Laboratory Medicine, The Robert Larner, M.D. College of Medicine, University of Vermont, Burlington, Vermont, USA.; 4 Department of Medicine,; 5 Cardiovascular Health Research Unit, and; 6 Department of Epidemiology, University of Washington, Seattle, Washington, USA.; 7 Divison of Cardiology, Department of Medicine, Johns Hopkins University, Baltimore, Maryland, USA.; 8 College of Pharmacy, University of Manitoba, Winnipeg, Manitoba, Canada.; 9 Department of Biochemistry, The Robert Larner, M.D. College of Medicine, University of Vermont, Burlington, Vermont, USA.; 10 Department of Health Services, School of Public Health, University of Washington, Seattle, Washington, USA.

**Keywords:** Cardiology, Immunology, Heart failure, Innate immunity

## Abstract

**Background:**

Immunomodulatory therapy may help prevent heart failure (HF). Data on immune cells and myocardial remodeling in older adults with cardiovascular risk factors are limited.

**Methods:**

In the Multi-Ethnic Study of Atherosclerosis cohort, 869 adults had 19 peripheral immune cell subsets measured and underwent cardiac MRI during the baseline exam, of which 321 had assessment of left ventricular global circumferential strain (LV-GCS). We used linear regression with adjustment for demographics, cardiovascular risk factors, and cytomegalovirus serostatus to evaluate the cross-sectional association of immune cell subsets with left ventricular mass index (LVMI) and LV-GCS.

**Results:**

The average age of the cohort was 61.6 ± 10.0 years and 53% were women. Higher proportions of **γ**/**δ** T cells were associated with lower absolute (worse) LV-GCS (–0.105% [95% CI –0.164%, –0.046%] per 1 SD higher proportion of **γ**/**δ** T cells, **P** = 0.0006). This association remained significant after Bonferroni’s correction. Higher proportions of classical monocytes were associated with worse absolute LV-GCS (–0.04% [95% CI –0.07%, 0.00%] per 1 SD higher proportion of classical monocytes, **P** = 0.04). This did not meet significance after Bonferroni’s correction. There were no other significant associations with LV-GCS or LVMI.

**Conclusion:**

Pathways associated with **γ**/**δ** T cells may be potential targets for immunomodulatory therapy targeted at HF prevention in populations at risk.

**Funding:**

Contracts 75N92020D00001, HHSN268201500003I, N01-HC-95159, 75N92020D00005, N01-HC-95160, 75N92020D00002, N01-HC-95161, 75N92020D00003, N01-HC-95162, 75N92020D00006, N01-HC-95163, 75N92020D00004, N01-HC-95164, 75N92020D00007, N01-HC-95165, N01-HC-95166, N01-HC-95167, N01-HC-95168, and N01-HC-95169 and grant R01 HL98077 from the National Heart, Lung, and Blood Institute/NIH and grants KL2TR001424, UL1-TR-000040, UL1-TR-001079, and UL1-TR-001420 from the National Center for Advancing Translational Sciences/NIH.

## Graphical abstract

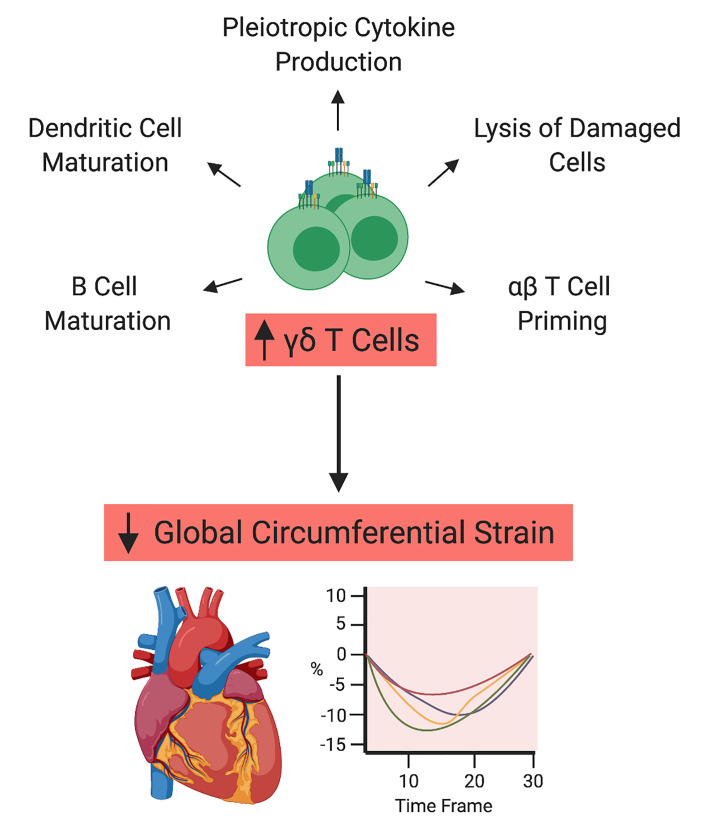

## Introduction

In adults without heart failure (HF), higher levels of circulating proinflammatory cytokines, such as TNF-α, IL-6, and C-reactive protein, have been associated with future HF events (1–3). However, targeting these inflammatory cytokines in adults who have developed HF has not been successful in slowing disease progression (4, 5). Secondary analysis of the Canakinumab Anti-Inflammatory Thrombosis Outcome Study showed a significant trend toward decreased HF events with IL-1β blockade in patients without HF but with prior myocardial infarction (MI; ref. 6). Thus, immunomodulatory therapies may be better suited for HF prevention in individuals at risk for HF and/or with subclinical cardiac mechanical impairment. Investigating the associations of innate and adaptive immune cells, integral in regulating the inflammatory milieu, with cardiac structure and function in older adults with cardiovascular risk factors but without HF can provide important insights into potential immune targets for HF prevention. In this study, we leveraged data obtained from a case-cohort substudy of the Multi-Ethnic Study of Atherosclerosis (MESA) evaluating peripheral blood mononuclear cells (PBMCs) and MI to investigate associations of innate and adaptive immune cell subsets with measures of cardiac structure and function on cardiac magnetic resonance imaging (CMR).

Due to limited data in humans without clinical disease, we examined a broad set of immune cell subsets. Nevertheless, our a priori hypotheses were guided by findings from experimental models of HF and clinical studies in humans with HF or broad cardiovascular disease (CVD). Proliferation and recruitment of monocytes from the circulation mediate cardiac hypertrophy and systolic dysfunction in mouse models (7–13). In humans, monocytes are categorized into classical, intermediate, and nonclassical subsets based on expression of CD14 (lipopolysaccharide) and CD16 (Fcγ III) receptors. Classical monocytes (CD14^++^CD16^–^) express inflammatory cytokines while intermediate monocytes (CD14^++^CD16^+^) may have inflammation-regulating effects (14–16). Impacts of other innate immune cell subsets, such as natural killer (NK) and γ/δ T cells, on cardiac dysfunction have not been as thoroughly studied. NK cells are thought to have a regulatory function and may be protective while there is experimental evidence that γ/δ T cells promote myocardial inflammation (17–19).

The adaptive immune system also plays an important role in myocardial remodeling (20–22). T cells, broadly categorized into CD4^+^ and CD8^+^ T cells, can be subdivided into regulatory and effector subsets. CD4^+^ T regulatory cells normally have a beneficial modulatory effect but can become increasingly dysfunctional in HF models, adopting a Th1 phenotype (23). Activation of naive (CD45RA^+^) T cells results in clonal expansion with differentiation into effector subtypes, such as cytotoxic CD8^+^ T cells. For CD4^+^ T cells, effector subtypes can include proinflammatory T helper 1 (Th1) and T helper 17 (Th17) cells as well as profibrotic T helper 2 (Th2) cells. Each subset is elevated in circulation and in the myocardium of HF animal models (24, 25). In murine ischemic HF, there is polarization toward a Th2 and Th17 phenotype with reversal of cardiac hypertrophy and systolic dysfunction after inhibition of CD4^+^ T cells (24). Subsequently, these activated T cells give rise to a pool of antigen-specific memory (CD45RO^+^) and terminally differentiated effector memory (TEMRA) T cells. Higher ratio of memory to naive T cells has been associated with atherosclerosis (26). With aging, there are also rising subpopulations of senescent (CD28^–^) T cells that are associated with comorbidities such as hypertension, diabetes, and atherosclerosis (27–30).

Given the consistent effects observed on cardiac hypertrophy and systolic function in experimental models, left ventricular mass index (LVMI) and left ventricular global circumferential strain (LV-GCS), a more sensitive measure of systolic function, were chosen as the primary outcomes. Both are also early imaging markers of cardiac remodeling and dysfunction that independently predict incident HF (31, 32). Our a priori hypotheses, based on studies described above, were (a) higher circulating proportions of classical monocytes, γ/δ T cells, and Th1 and Th17 cells are associated with worse cardiac structure and function (higher LVMI and lower absolute LV-GCS); and (b) higher circulating proportions of intermediate monocytes, NK cells, and regulatory T cells (Tregs) are associated with better cardiac structure and function (lower LVMI and higher absolute LV-GCS).

## Results

**Figure 1:**
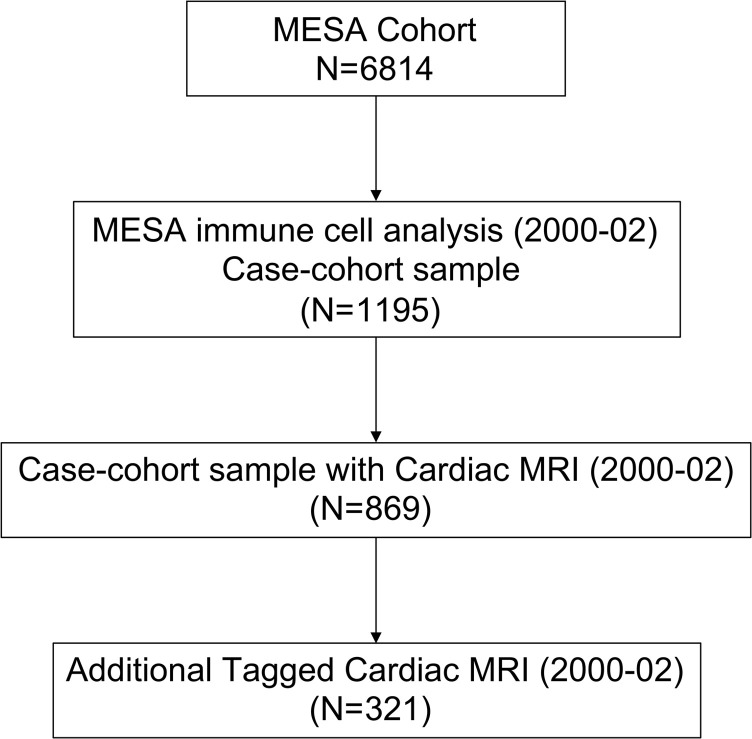
MESA study design with immune cell subset case-cohort and cardiac MRI.

Out of 1195 MESA participants who underwent immune cell phenotyping, 869 participants had CMR performed. The mean proportions of each immune cell subset are included in Supplemental Table 2; supplemental material available online with this article; https://doi.org/10.1172/jci.insight.149193DS1 The distributions of these cells were similar to those measured from fresh whole blood samples obtained during MESA Exam 4 (33). The baseline characteristics of these participants are included in Table 1. The average age of the cohort was 61.6 ± 10.0 years with approximately 53% women and 28% Blacks. In addition, 321 participants also had tagged CMR for assessment of circumferential strain (Figure 1).

Our a priori hypotheses evaluated the associations of classical monocytes (CD14^++^CD16^–^), intermediate monocytes (CD14^+^CD16^+^), and NK, γ/δ, Th1 (CD4^+^IFN^+^), Th17 (CD4^+^IL-17^+^), and regulatory (CD4^+^CD25^+^CD127^–^) T cells with LV-GCS and LVMI. Absolute strain values were used for ease of interpretation, with a lower absolute strain value representing less systolic deformation and worse function. As shown in Table 2, higher proportions of γ/δ T cells were associated with lower absolute (worse) LV-GCS (–0.11% [95% CI –0.16%, –0.05%] per 1 SD higher proportion of γ/δ T cells, *P* = 0.0006). This association remained significant even after correction for multiple testing. Higher proportions of classical monocytes were associated with worse LV-GCS (–0.04% [95% CI –0.07%, 0.00%] per 1 SD higher proportion of classical monocytes, *P* = 0.04). However, this association was not significant after Bonferroni’s correction. None of the other immune cell subsets selected a priori had a significant association with LV-GCS. There were also no significant associations among the remaining immune cell subsets and LV-GCS. Of note, the association of Th2 CD4^+^ T cells with LV-GCS (0.25% [95% CI –0.01%, 0.50%] per 1 SD higher proportion of Th2 CD4^+^ T cells, *P* = 0.06) had the largest effect size of all the immune cell subsets but did not meet statistical significance. As shown in Table 3, there were no significant associations between immune cell subsets and LVMI.

In addition, we performed prespecified analyses evaluating for interaction of immune cell subsets with sex and blood pressure with respect to LV-GCS. We observed a significant interaction between sex and classical monocytes but not γ/δ T cells (Supplemental Table 3). In stratified analysis, higher proportions of classical monocytes were associated with worse LV-GCS (–0.09% [95% CI –0.13%, –0.05%] per 1 SD higher proportion of classical monocytes, *P* = 0.0001) in women (Table 4). There was no significant association observed between classical monocytes and LV-GCS in men. We further created estimated marginal plots to visualize the effect of sex on the associations between the immune cell subsets and LV-GCS. As illustrated in Supplemental Figure 6A, there was an inverse association between proportions of classical monocytes and LV-GCS in women while there was no significant association in men. Sex did not modify the association between γ/δ T cells and LV-GCS (Supplemental Figure 6B).

The associations of γ/δ T cells and classical monocytes with LV-GCS had significant interaction with hypertension status (Supplemental Table 4). In stratified analysis, higher proportions of γ/δ T cells were associated with worse LV-GCS (–0.17% [95% CI –0.27%, –0.07%] per 1 SD higher proportion of γ/δ T cells, *P* = 0.0017) in adults without hypertension (Table 5). Similarly, higher proportions of classical monocytes were associated with worse LV-GCS (–0.10% [95% CI –0.15%, –0.05%] per 1 SD higher proportion of classical monocytes, *P* = 0.0003) in adults without hypertension (Table 5). There was no significant association observed between γ/δ T cells or classical monocytes and LV-GCS in adults with hypertension. The estimated marginal plots further illustrate an inverse association of higher proportions of classical monocytes and γ/δ T cells with LV-GCS in participants without hypertension but no clear association in those with hypertension (Supplemental Figure 7, A and B). In order to better understand the effect of antihypertensive medications, we further stratified the hypertensive group by treatment status (Table 5). In adults with untreated hypertension, higher proportions of γ/δ T cells were associated with worse LV-GCS (–0.14% [95% CI –0.26%, –0.02%] per 1 SD higher proportion of γ/δ T cells, *P* = 0.04). But no association was observed between γ/δ T cells and LV-GCS in those on antihypertensive medications (–0.04% [95% CI –0.10%, 0.03%] per 1 SD higher proportion of γ/δ T cells, *P* = 0.28). In contrast, classical monocytes did not have an association with LV-GCS in adults with hypertension regardless of treatment status.

## Discussion

In a multicenter cohort of middle-aged adults free of CVD, who had PBMCs collected and CMR performed during the same period, we observed a significant association between higher proportions of γ/δ T cells and worse cardiac function, as measured by absolute LV-GCS (Table 2). The association with γ/δ T cells was significantly modified by antihypertensive medication use (Table 5). While the association of higher proportions of classical monocytes with LV-GCS did not meet significance after Bonferroni’s correction, there was a significant interaction (Tables 4 and 5) with sex and hypertension status. There was no significant association observed between any of the immune cell subsets and LVMI. These findings highlight specific immune cell subsets that may play a role in adverse cardiac remodeling in older adults with cardiovascular risk factors but without HF.

Our findings related to γ/δ T cells add to prior experimental results demonstrating the detrimental effects of γ/δ T cells in different types of cardiomyopathies. In our cohort, γ/δ T cells represented on average 6.7% of all CD3^+^ T cells in the peripheral blood. Unlike MHC-restricted αβ CD4^+^ T cells, a majority of γ/δ T cells are activated in an MHC-independent manner by a broad set of antigens (34). The capacity of γ/δ T cells to recognize antigens displayed by damaged tissue and rapidly respond within hours without requiring clonal expansion allows them to participate in immunosurveillance, referred to as lymphoid stress surveillance (35, 36). Thus, γ/δ T cells can perform a broad set of functions from creating a local effector response to triggering the adaptive immune system (36). Prior mechanistic studies have illustrated that γ/δ T cells promote infiltration of neutrophils and macrophages into the myocardium via pathways involving IL-17, IL-1β, and upregulation of the pathogenic Th1 response (18, 37, 38). We also observed that the association of γ/δ T cells with LV-GCS was significantly mitigated in individuals on antihypertensive medications, suggesting potential immunomodulatory effects. Comparisons of our findings with experimental models are inherently limited because immune cell subsets and pathways may markedly change when transitioning from a preclinical or subclinical state (captured by our cohort) to a disease state (captured by experimental models). Thus, our results are hypothesis generating and can only be extrapolated to adults without clinical HF. Further studies are needed to determine if higher proportions of γ/δ T cells reflect a state of dysregulated immunosurveillance that contributes to progressive adverse myocardial remodeling in adults with cardiovascular risk factors.

We also observed an inverse association between higher proportions of classical monocytes and LV-GCS that was significant at the *P* value threshold of less than 0.05 but not after Bonferroni’s correction. However, there was a strong interaction with sex, as there was a significant association between higher proportions of classical monocytes and LV-GCS in women but not in men (Supplemental Figure 6A). Classical monocytes are the predominant monocyte subtype in humans, making up roughly 85% of all circulating monocytes (39). They infiltrate injured tissue and differentiate into monocyte-derived macrophages and dendritic cells with the ability to either augment tissue damage or promote tissue regeneration and healing (40). Disturbing this delicate balance can alter the fate of the injured tissue. Experimental studies have demonstrated an association between classical monocytes and cardiac dysfunction in ischemic models (41, 42). In humans, peripheral monocytosis in adults with MI is associated with greater cardiac dysfunction, and adults with heart failure with preserved ejection fraction (HFpEF) also have higher levels of peripheral monocytes (43–46). However, it is important to note that our cohort did not include individuals with CVD, and thus the pathways regulating different immune cell subsets are likely different in our cohort compared with experimental models and cohorts, including individuals with existing CVD. There is evidence suggesting sex-related differences in monocytes. Monocyte levels increase with age in women but not in men, and animal models have demonstrated greater macrophage activation with phagocytic and inflammatory activity in female animals (47–50). While estrogen levels decrease after menopause, as would be expected in our relatively older cohort, there is evidence that sex-specific gene regulation can occur in the absence of circulating sex hormones (51). Thus, the relationship observed here between classical monocytes and worse cardiac function in women merits further investigation. Future studies should investigate if and how classical monocytes may play a role in progression from subclinical disease to clinical HF, especially HFpEF, in women with cardiovascular risk factors, given the rising incidence of HFpEF in women (52).

### Study limitations.

Our study had important limitations. First, this is a cross-sectional study. Thus reverse causality cannot be ruled out, although it is unlikely given the individuals in the cohort did not have clinical HF at the time of PBMC collection and CMR scanning. Future longitudinal studies are needed to account for intraindividual variability and understand the changes in immune cell subsets that occur over time with aging and accrual of cardiovascular comorbidities. Furthermore, better characterization of tissue-resident leukocytes is needed because peripheral immune cells may not be representative of myocardial resident immune cells, especially in the absence of an acute insult (53). Second, as with any observational study, there may be residual confounding, even though we adjusted for important potential confounders in our models. Third, only a subgroup of the participants with immune cell subset data underwent CMR, and a smaller subgroup received a tagged CMR study to evaluate for GCS. The limited sample size may have introduced selection bias and reduced power. However, the selection bias was likely minimal because the participants were largely chosen at random for the primary CMR and the tagged CMR studies. Last, participants were chosen for immune cell subset measurement using a case-cohort design, thus weighting the cohort toward individuals who went on to develop CVD. We adjusted for this by using sampling weights to minimize the bias of the estimates.

### Conclusions.

We observed a significant association between higher proportions of γ/δ T cells and worse cardiac function as measured by LV-GCS. This finding in an older population-based cohort with cardiovascular risk factors but without HF provides insight into which immune cell subsets may contribute to subclinical cardiac dysfunction even in the absence of acute myocardial injury. Our findings suggest that pathways associated with γ/δ T cells and classical monocytes may be potential targets for immunomodulatory therapy targeted at HF prevention in similar populations with cardiovascular risk factors. Future mechanistic and longitudinal studies should evaluate the role of γ/δ T cells in development of cardiac dysfunction and HF.

## Methods

### Study cohort.

The MESA is a multicenter, population-based cohort comprising 6814 men and women aged 45 to 84 years at baseline, recruited from 6 field centers in the United States. Exclusion criteria included active treatment for cancer, amputation, pregnancy, or a history of clinical CVD, including MI, angina, cardiovascular procedures, HF, and/or cerebrovascular disease. Participants were interviewed and examined at the baseline exam (2000–2002). Participants in MESA self-identified as White, Black, Hispanic, or Chinese, and additional details on the design of the MESA cohort have been described previously (54). As part of a case (participants with coronary heart disease)–cohort study, immune cell phenotypes were obtained on 1195 MESA participants from PBMCs obtained and stored at baseline (55). For this study, we leveraged this immune cell phenotyping data to determine cross-sectional associations with cardiac structure and function obtained through CMR performed at baseline. Although a case-cohort design may reduce power for the primary question of association with MI, the design uses sampling weights to obtain valid estimates of associations with other phenotypes and thus provides opportunities for additional studies such as this one (56).

### Clinical data.

Clinical covariate data collected during the baseline exam in 2000 to 2002 were used. Standardized questionnaires were used to obtain demographics, past medical history, medications, and smoking status. Smoking was defined as never, former (no cigarettes within the past 30 days), or current. Trained clinical staff measured the weight, height, and resting blood pressure of each participant. Fasting lipid profile and serum creatinine were also obtained for each participant. IgG antibodies against CMV were measured by enzyme immunoassay (Diamedix Corp). The interassay coefficients of variation were 5.1% to 6.8% for the CMV IgG immunoassay.

### Immune cell subset phenotyping.

Immune cell phenotyping performed in this cohort has been previously described in detail (55, 57–59). PBMCs were isolated from blood collected in 8 mL citrate CPT tubes (BD Biosciences) during the MESA baseline exam in 2000 to 2002. The isolated PBMCs were washed and cryopreserved in media containing 90% fetal bovine serum and 10% dimethylsulfoxide at –135°C. At the time of phenotyping (2016), the cells were thawed at 37°C for 15 minutes and treated with benzonase (MilliporeSigma, 250 U/mL in RPMI from Invitrogen, Thermo Fisher Scientific) for 20 minutes. Cells were slowly diluted 10-fold by the addition of RPMI supplemented with fetal bovine serum (10%), l-glutamine (2 mM), penicillin (100 IU/mL), and streptomycin (100 μg/mL) (fsRPMI) with mixing. Cells were pelleted by centrifugation (5 minutes at 200*g* at room temperature) and treated with 250 U/mL benzonase in fsRPMI for 10 minutes. The pellet was washed and filtered through a 70 μm filter.

For surface labeling, cells were incubated with LIVE/DEAD stain (Thermo Fisher Scientific catalog L34955 or L34957) for 15 minutes in PBS, pH 7.4. The viability stain was removed by centrifugation for 5 minutes at 200*g* at room temperature, and antibodies or isotype-matched controls were added as detailed in Supplemental Table 1 and incubated for 15 minutes at room temperature in the dark. Excess antibody was removed by centrifugation, the cells were washed, and the final pellet was placed in 1% paraformaldehyde and stored refrigerated until flow cytometry was performed. All antibodies were from Miltenyi Biotec and used at manufacturer-recommended dilutions. Preliminary testing indicated that the inclusion of Fc block in this system did not significantly alter the percentages of the measured cell types, and so Fc block was not included.

For intracellular staining assays (Th1, Th2, and Th17), PBMCs were activated with phorbol myristic acid/ionomycin in the presence of brefeldin A, as previously described (33). After incubating for 3 hours at 37°C, 5% CO_2_, cells were centrifuged, resuspended in PBS at pH 7.4, incubated with a LIVE/DEAD stain (Thermo Fisher Scientific catalog L34955) for 15 minutes at room temperature, and then centrifuged at 300*g* for 5 minutes at room temperature. The PBMCs were resuspended in CD4/CD8 antibodies or appropriate isotypes and incubated for 15 minutes at room temperature in the dark. Samples were centrifuged and the pellets were washed and fixed with 2% paraformaldehyde (Alfa Aesar catalog 43368) for 10 minutes. Paraformaldehyde was removed by centrifugation for 5 minutes at 200*g* at room temperature, and cells were incubated with antibodies against IL-4, IL-17, and IFN-γ in the presence of 1% saponin (MilliporeSigma). After 15 minutes, cells were washed and resuspended in paraformaldehyde until flow cytometry was performed.

Flow cytometry was performed on an MQ10 flow cytometer and analyzed with MACS Quantify software (Miltenyi Biotec). Calibration beads were used for daily calibration. Compensation was set using single-color compensation controls, and isotype controls were used to set negative gates for each assay. Cell phenotypes were expressed as percentages. Flow cytometry gating strategies are presented in Supplemental Figures 1–5. All data are presented as percentages (CD4 subsets as % CD4^+^ T cells, CD8 subsets as % CD8^+^ T cells, monocyte subsets as % CD14^+^ monocytes, γ/δ T cells as % CD3^+^ T cells, and NK cells as % gated lymphocytes). Percentages, instead of absolute numbers, were used since PBMCs for this cohort were cryopreserved for many years. Due to sample manipulation from cryopreservation, our internal analyses have demonstrated percentages, rather than absolute counts, to be a better representation of circulating cells in the whole blood.

### Cardiac MRI.

CMR was performed in 5098 participants during the MESA baseline exam in 2000 to 2002. Of these, 1773 individuals were randomly selected to undergo tagged CMR for LV-GCS measurement as an ancillary study protocol during the baseline exam. In our study sample, 869 of the 1195 participants included in the case-cohort sample underwent CMR, of whom 321 also had a tagged CMR. The 2 primary CMR outcomes were LVMI and LV-GCS. MRI data were acquired using 1.5 T scanners (General Electric and Siemens). The CMR protocol has been described previously (60, 61). Briefly, 2- and 4-chamber cine MRI series were acquired after standard scout imaging. Short-axis cine images were acquired from above the mitral valve to the left ventricular (LV) apex with retrospective gating. Three tagged short-axis slices from LV base to apex were obtained using an electrocardiographically triggered, segmented, *k*-space fast spoiled gradient-echo pulse sequence. The endocardial and epicardial borders were contoured using a semiautomated method (MASS 4.2, Medis). The difference between the epicardial and endocardial areas for each slice at end diastole was multiplied by the slice thickness, which was then multiplied by the density of the myocardium to determine the LV mass. Papillary muscle mass was excluded from the LV mass. LV mass was indexed to the body surface area. Short-axis-tagged slices were analyzed using the HARP method (MATLAB, MathWorks, or HARP1.15, Diagnosoft). Circumferential strain was calculated for 4 segments (anterior, posterior, lateral, and septal) from each of the 3 short-axis slices. LV-GCS describes circumferential shortening during systole and thus is a negative value, with more negative values denoting better function (62). LV-GCS had excellent intraobserver, interobserver, and interstudy reproducibility, with intraclass correlation coefficients ranging from 0.7 to 0.9 (62, 63). Segments with significant noise were excluded from the analysis (7.9%). LV-GCS was calculated by averaging the existing segments. Absolute LV-GCS values are reported for ease of interpretation.

### Statistics.

Linear regression was used to determine the association between the proportions of immune cell subsets and the CMR parameters. Given the case-cohort design, sampling weights were incorporated into the regression model to obtain unbiased estimates, and robust SEM estimates were used. One model was used for each immune cell subset (independent variable) and CMR parameter (dependent variable). The models were adjusted for potential confounders, which included baseline age, sex, race/ethnicity, smoking status, BMI, diabetes status, SBP, total cholesterol, HDL-cholesterol, baseline statin use, eGFR, and log-transformed CMV serostatus. As part of our a priori analytic plan, we evaluated potential interactions with sex and hypertension, defined as SBP at least 140 mmHg or use of antihypertensive medications, in immune cell subsets that had a significant association with either of the CMR outcomes. If the interaction term was significant, we performed stratified analyses and created estimated marginal plots to visually display how sex or hypertension modified the association between the immune cell subset and the CMR outcome. Bonferroni’s correction was used to account for multiple testing. For the 7 immune cell subsets included in the a priori hypotheses, *P* value threshold was set at ≤0.003 (14 tests). For the remaining 12 immune cell subsets, *P* value threshold was set at ≤0.001 (38 total tests).

### Study approval.

All participants provided written informed consent for participation in the study, and all procedures were conducted under institutionally approved protocols for human subject research. The study protocol was approved by the IRBs of the coordinating center (University of Washington, Seattle, Washington, USA) and each of the MESA field centers.

## Author contributions

AS designed the study and wrote the manuscript. ASR, CS, AF, and JAD analyzed the data and edited the manuscript. MFD, SAH, and NCO conducted the experiments and edited the manuscript. JACL acquired the data and edited the manuscript. MFD, SJS, RPT, and BMP designed the study and edited the manuscript.

## Supplementary Material

Supplemental data

Trial reporting checklists

ICMJE disclosure forms

## Figures and Tables

**Table 1:**
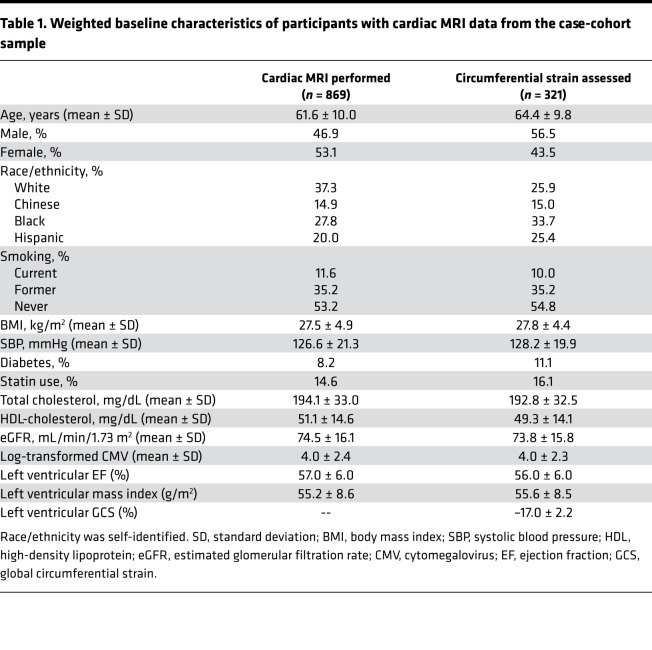
Weighted baseline characteristics of participants with cardiac mri data from the case-cohort sample

**Table 2:**
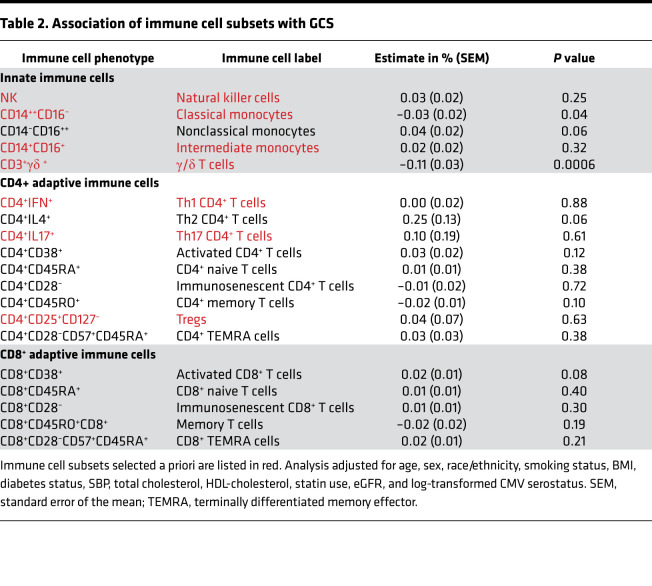
Association of immune cell subsets with GCS

**Table 3:**
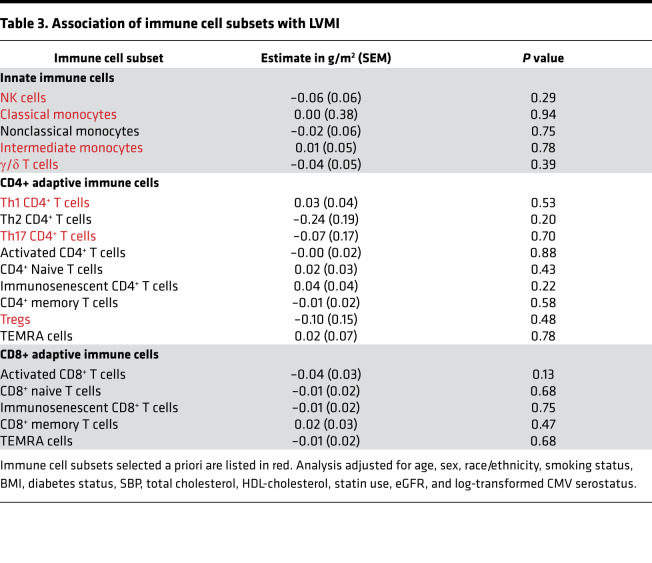
Association of immune cell subsets with LVMI

**Table 4:**
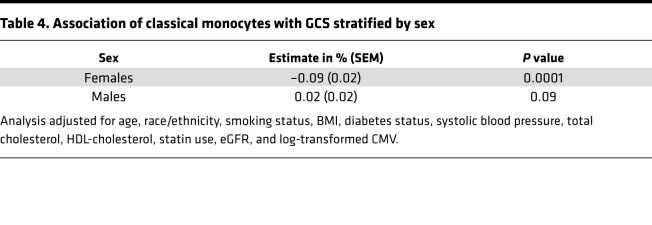
Association of classical monocytes with GCS stratified by sex

**Table 5:**
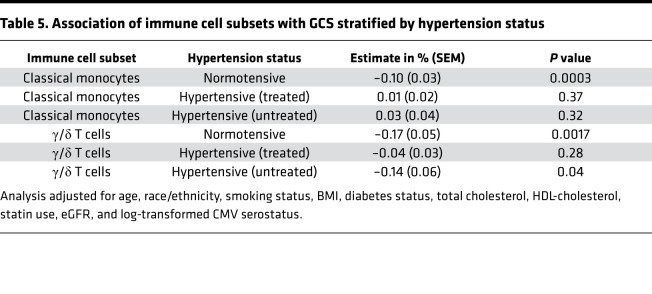
Association of immune cell subsets with GCS stratified by hypertension status

## Version 1 07/08/2021

Electronic publication
