# Supplementary material for: Association of immune cell subsets with cardiac mechanics in the Multi-Ethnic Study of Atherosclerosis
Source: JCI Insight. 2021 Jul 8;6(13):e149193. doi: 10.1172/jci.insight.149193 (PMC8410049; doi:10.1172/jci.insight.149193)
Supplement: Supplemental data [file jciinsight-6-149193-s061.pdf]

Supplemental Materials

Supplemental Table 1: Flow Cytometry Antibody Isotypes and Fluorophores (Miltenyi Biotec).

Assay	Antigen	Fluorophore	Antibody	Isotype
**CD4 Subtypes**	CD4	Per-CP-Vio	130-103-793	130-097-561
	CD45RA	APC-Vio	130-096-604	130-096-822
	CD45RO	VioBlue	130-099-044	130-094-671
	CD28	PE	130-092-921	130-092-212
	CD38	PE-Vio	130-099-151	130-096-638
	CD57	APC	130-092-141	130-093-176
	CD27	VioBright FITC	130-104-845	130-104-513
**CD8 Subtypes**	CD8	Per-CP-Vio	130-097-911	130-097-563
	CD45RA	APC-Vio	130-096-604	130-096-822
	CD45RO	VioBlue	130-099-044	130-094-671
	CD28	PE	130-092-921	130-092-212
	CD38	PE-Vio	130-099-151	130-096-638
	CD57	APC	130-092-141	130-093-176
	CD27	VioBright FITC	130-104-845	130-104-513
**γδT Cells NK Cells**	CD16	Per-CP-Vio	130-100-430	130-098-595
	CD3	VioBlue	130-094-363	130-094-671
	CDγδ TCR	PE	130-096-869	130-092-212
	CD56	PE-Vio	130-100-676	130-104-616
**Monocyte Subtypes**	CD16	Per-CP-Vio	130-100-430	130-098-595
	TIE-2	APC-Vio	130-101-607	130-104-618
	CX3CR1	PE	130-105-837	130-104-612
	CD14	PE-Vio	130-096-628	130-096-638
	HLA-DR	APC	130-095-297	130-091-836
	CD163	FITC	130-096-626	130-092-213
**Regulatory T Cells**	CD127	PeVio	130-099-719	130-096-638
	CD25	VioBright FITC	130-104-274	130-104-575
	CD4	APC	130-092-374	130-096-653
**Th1/2/17 Cells**	CD4	APC	130-092-374	130-096-653
	CD8	VioBright FITC	130-104-519	130-104-512
	IFNγ	PE-Vio 770	130-096-752	130-113-202
	IL4	PE	130-092-212	130-091-647
	IL17	APC-Vio 770	130-096-656	130-096-653

Supplemental Table 2. Summary of Immune Cell Subsets in Participants with Cardiac MRI from the MESA Case-Cohort Sample

Immune Cell Phenotype	Immune Cell Label	N	% (Mean ± SD
**Innate Immune Cells**			
NK	Natural Killer Cells	1087	4.9 ± 5.6
CD14++CD16-	Classical Monocytes	922	74.6 ± 10.3
CD14-CD16++	Non-classical Monocytes	922	7.3 ± 7.3
CD14+CD16+	Intermediate Monocytes	922	18.1 ± 7.1
CD3+γδ+	Gamma-delta (γδ) T Cells	1087	6.7 ± 6.3
**CD4+ Adaptive Immune Cells**			
CD4+IFN+	Th1 CD4+ T Cells	770	15.0 ± 9.0
CD4+IL4+	Th2 CD4+ T Cells	770	2.9 ± 1.8
CD4+IL17+	Th17 CD4+ T Cells	770	2.1 ± 1.4
CD4+CD38+	Activated CD4+ T Cells	1051	26.7 ± 12.2
CD4+CD45RA+	CD4+ Naïve T Cells	1051	26.4 ± 11.8
CD4+CD28-	Immunosenescent CD4+ T Cells	1051	13.6 ± 9.9
CD4+CD45RO+	CD4+ Memory T Cells	1051	51.5 ± 13.3
CD4+CD25+CD127-	T Regulatory Cells	1035	5.0 ± 2.2
CD4+CD28-CD57+CD45RA+	TEMRA Cells	1051	5.4 ± 5.1
**CD8+ Adaptive Immune Cells**			
CD8+CD38+	Activated CD8+ T Cells	1062	23.7 ± 12.2
CD8+CD45RA+	CD8+ Naïve T Cells	1062	52.3 ± 14.3
CD8+CD28-	Immunosenescent CD8+ T Cells	1062	55.1 ± 15.9
CD8+CD45RO+	CD8+ Memory T Cells	1062	21.9 ± 10.3
CD8+CD28-CD57+CD45RA+	TEMRA Cells	1062	32.3 ±14.0

CD4 subsets are displayed as % overall CD4+ T cells, CD8 subsets are displayed as % overall CD8+ T cells, Monocyte subsets are displayed as % overall CD14+ monocytes, γδ T cells are displayed as % overall CD3+ T cells, NK cells are displayed as % gated lymphocytes.

SD: standard deviation
TEMRA: Terminally differentiated effector memory

Supplemental Table 3. Interaction of Sex with Immune Cell Subsets on Association with Global Circumferential Strain

Immune Cell Subset	Estimate (SE)	p-value
Classical Monocytes	**0.09 (0.02)**	**0.0001**
Gamma-delta (γδ) T Cells	0.05 (0.06)	0.43

Analysis adjusted for age, sex, race/ethnicity, smoking status, body-mass index, diabetes status, systolic blood pressure, total cholesterol, high density lipoprotein (HDL) cholesterol, statin use, estimated glomerular filtration rate, and log-transformed cytomegalovirus serostatus.

SE: standard error

Supplemental Table 4. Interaction of Hypertension with Immune Cell Subsets on Association with Global Circumferential Strain

**Immune Cell Subset**	**Estimate (SE)**	**p-value**
Classical Monocytes	**0.07 (0.02)**	**0.0001**
Gamma-delta (γδ) T Cells	**0.16 (0.07)**	**0.02**

Analysis adjusted for age, sex, race/ethnicity, smoking status, body-mass index, diabetes status, systolic blood pressure, total cholesterol, high density lipoprotein (HDL) cholesterol, statin use, estimated glomerular filtration rate, and log-transformed cytomegalovirus serostatus.

SE: standard error

Supplemental Figure 1. Gating Strategy for Regulatory T cells.

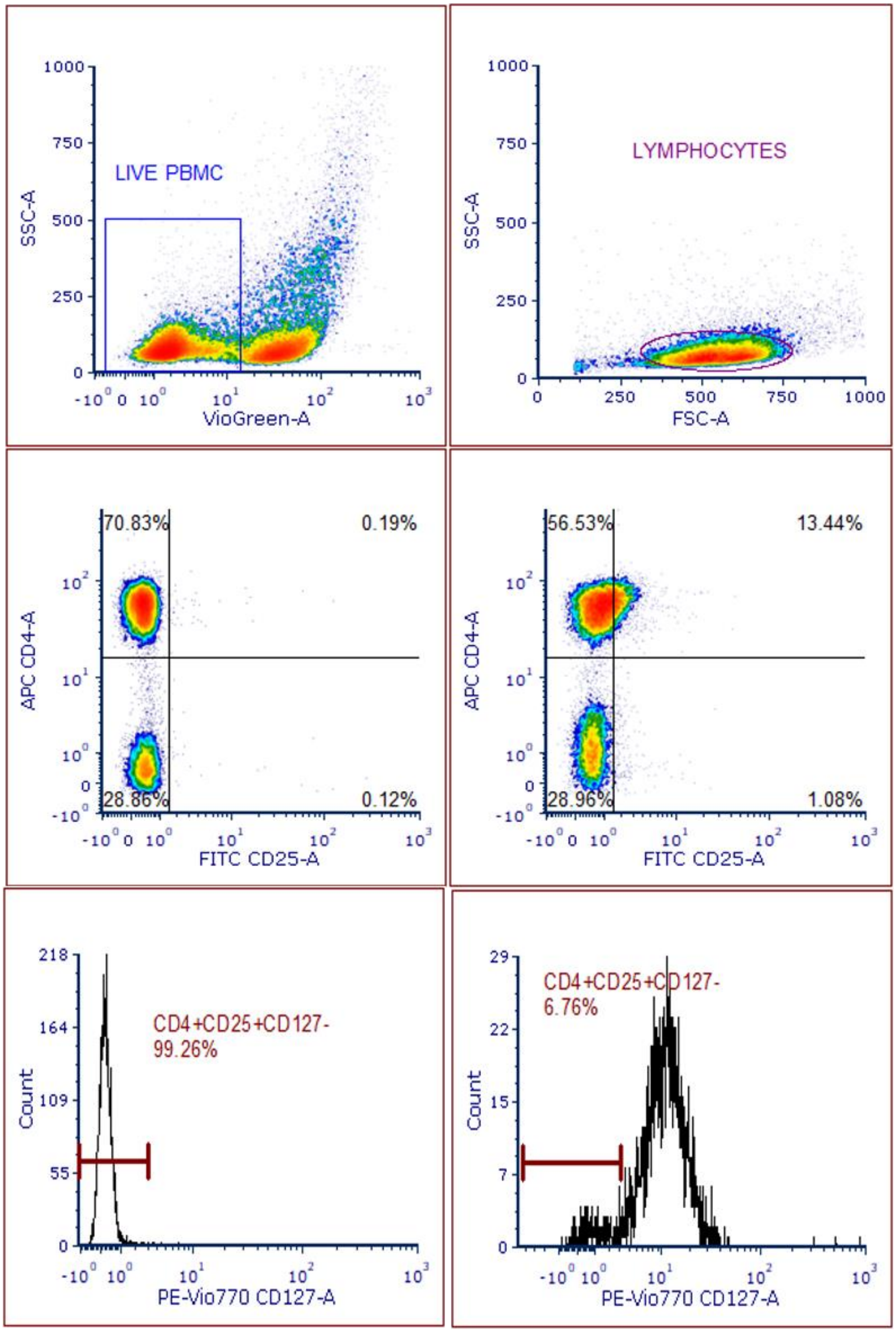

PBMCs were gated on live cells, then gated on lymphocytes, based on size and shape. Isotype controls were used to set negative gates using a CD4+ FMO tube. Histograms (Row 3) were gated on the CD4+CD25+ gate. Regulatory T cells are CD4+CD25+CD127-. Experiment conducted on 1035 samples. Original source: Feinstein MJ et al, ATVB 2021.

Supplemental Figure 2. Gating Strategy for γδ T cells and Natural Killer cells.

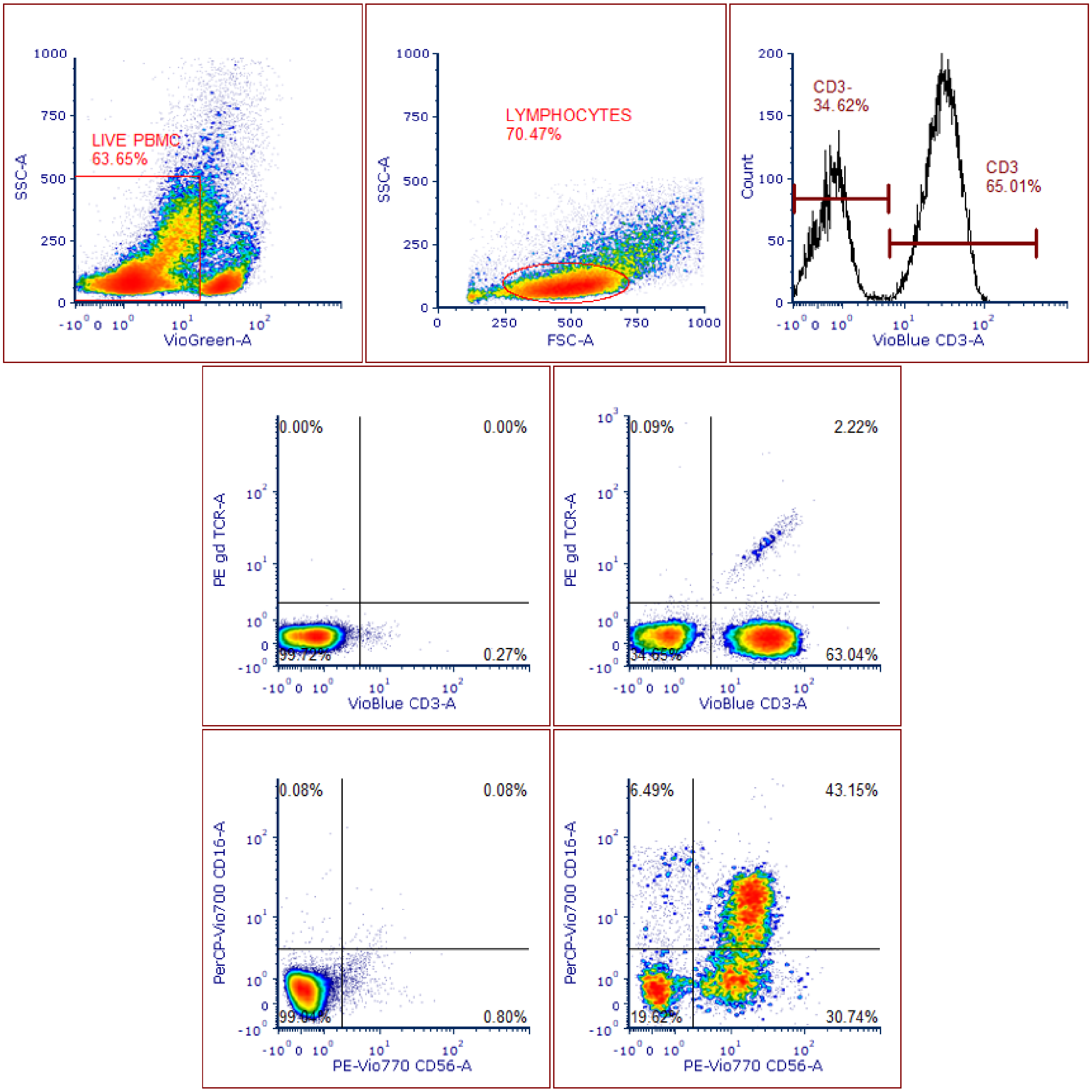

PBMCs were gated on live cells, then on lymphocytes based on size and shape. Isotype controls were used to set negative gates (2nd row, plot 1 and 3rd row plot 1). The lymphocyte gate was used to determine CD3+, CD3-, and γδ T cells (expressed as %CD3). NK cells were gated on the CD3- gate. Experiment conducted on 1087 samples. Original source: Feinstein MJ et al, ATVB 2021.

Supplemental Figure 3. Gating Strategy for CD4 and CD8 T Cell Subsets.

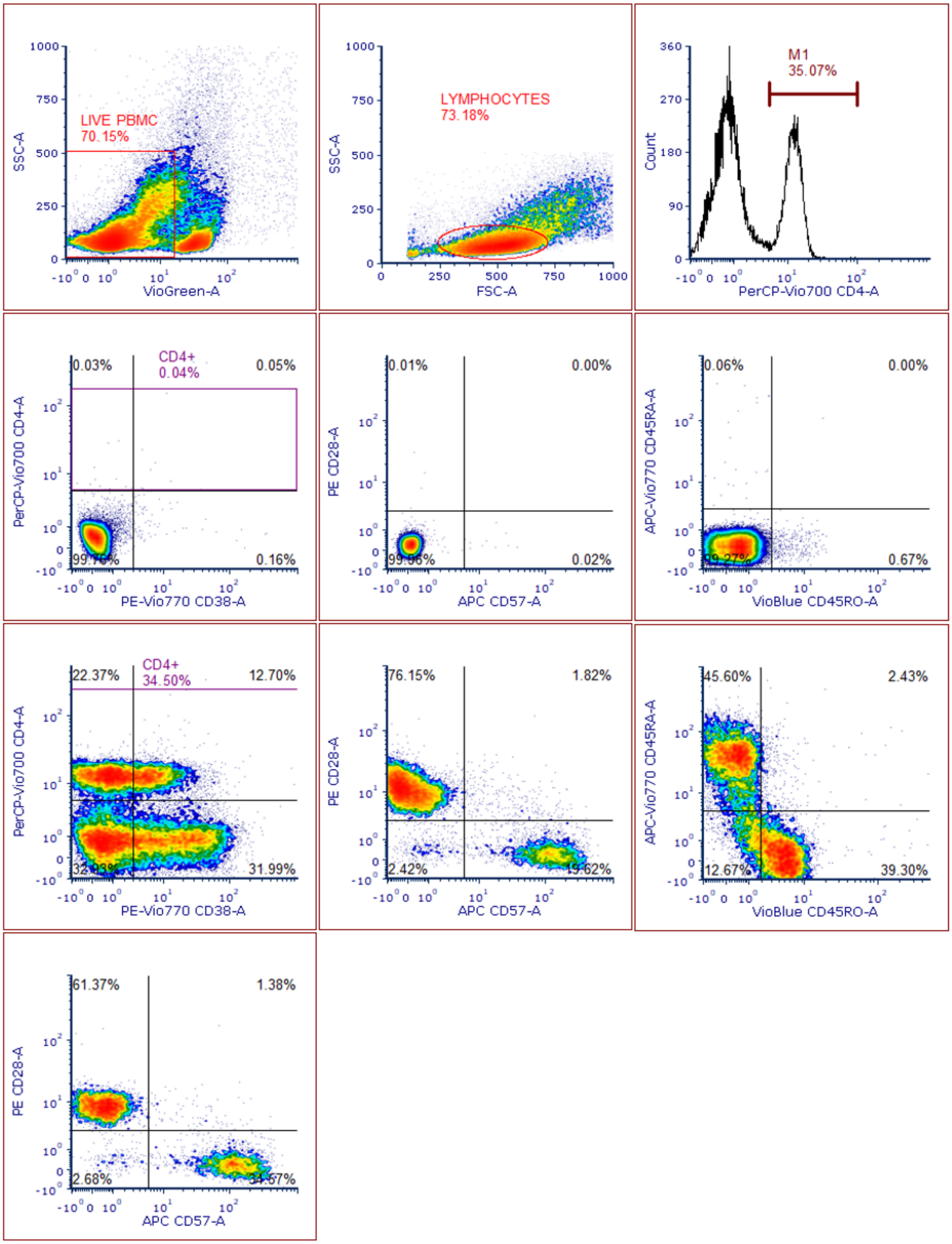

PBMCs were gated on live cells, then on lymphocytes based on size and shape. Isotype controls were used for negative gate setting (Row 2) and the test sample (3rd row). The 4th row was gated on the CD4+CD45RA+ gate to yield the CD4+ T_{EMRA} cells (CD4+CD45RA+CD28-CD57+). The CD8 samples were done identically except CD8 was substituted for CD4. Experiment conducted on 1051 and 1062 samples for CD4 and CD8 immune cell subsets, respectively. Original source: Feinstein MJ et al, ATVB 2021.

Supplemental Figure 4. Gating Strategy for Th1/2/17 Cell Subsets.

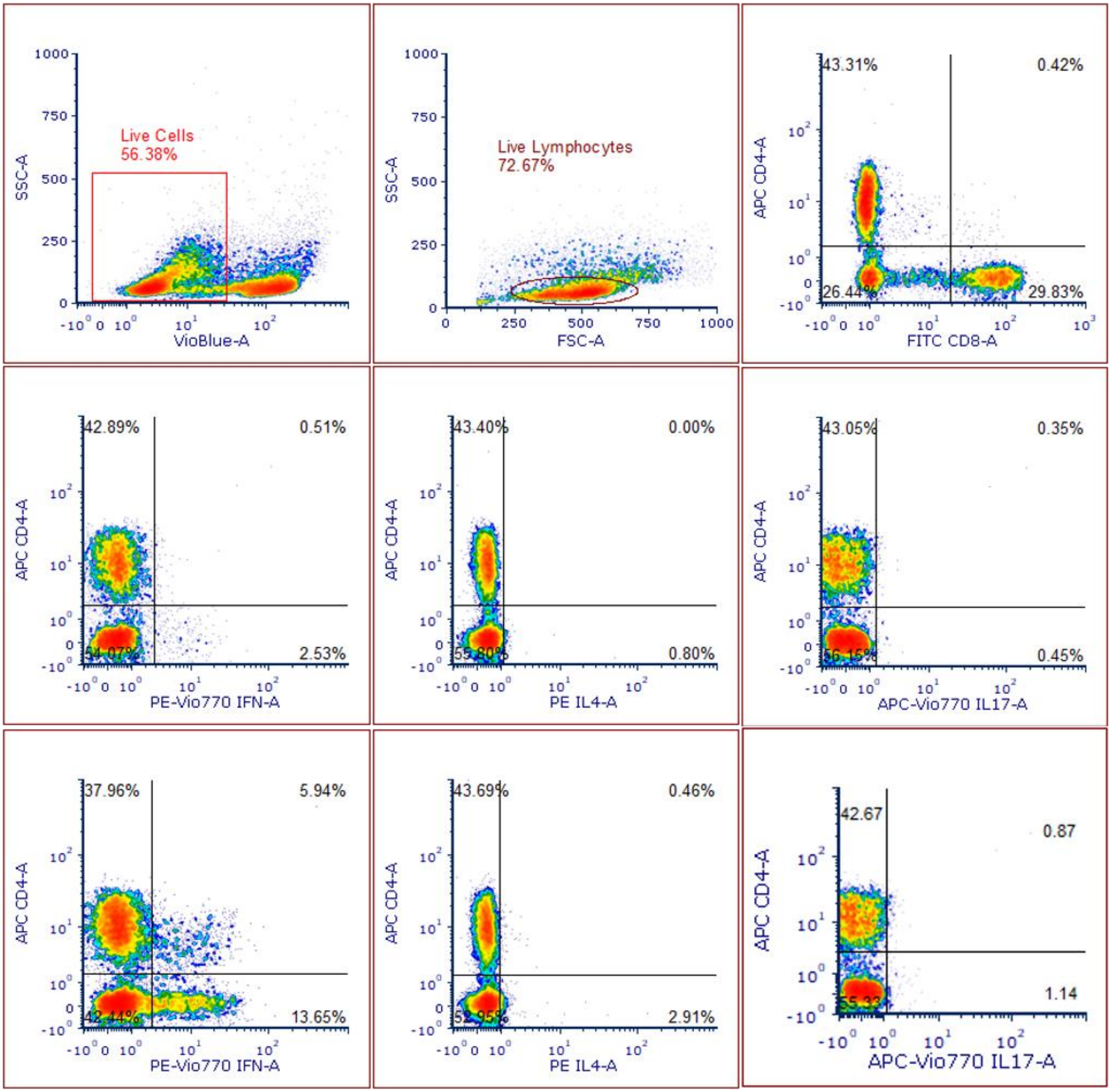

PBMCs were gated on live cells, then on lymphocytes based on size and shape (top row). Using a modified FMO approach (middle row), a CD4/CD8 tube with appropriate isotypes was used to set negative gates for IFNγ, IL4 and IL17. Finally, the test sample allows for the quantitation of IFNγ, IL4 and IL17 produced by the cells. Experiment conducted on 770 samples. Original source: Feinstein MJ et al, ATVB 2021.

Supplemental Figure 5. Gating Strategy for Monocyte Subsets.

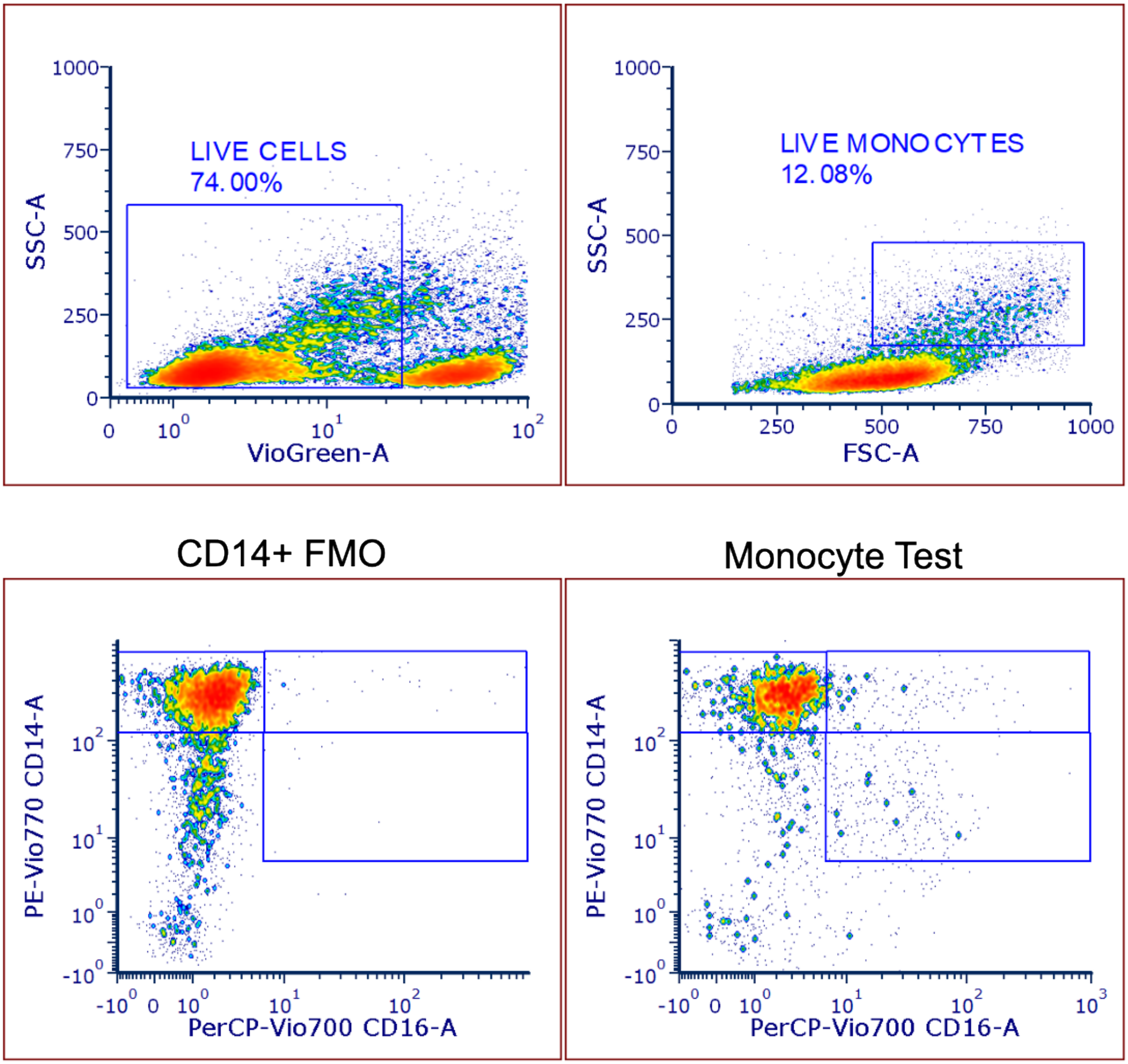

PBMCs were gated on live cells, then on monocytes based on size/shape. Using a CD14+ fluorescence minus one (FMO) approach, the negative CD16 gate was set. Finally, the CD16 Ab was added to the test sample to yield the CD14++CD16- gate, the CD14+CD16+ gate and the CD14dimCD16+ gate. These 3 gates are added to yield the CD14 total and the 3 gates are reported as percentage of this total CD14. Experiment conducted on 922 samples. Original source: Feinstein MJ et al, ATVB 2021.

Supplemental Figure 6. Estimated Marginal Plots Evaluating the Interaction of Sex on the Association between Immune Cell Subsets and Global Circumferential Strain

6A

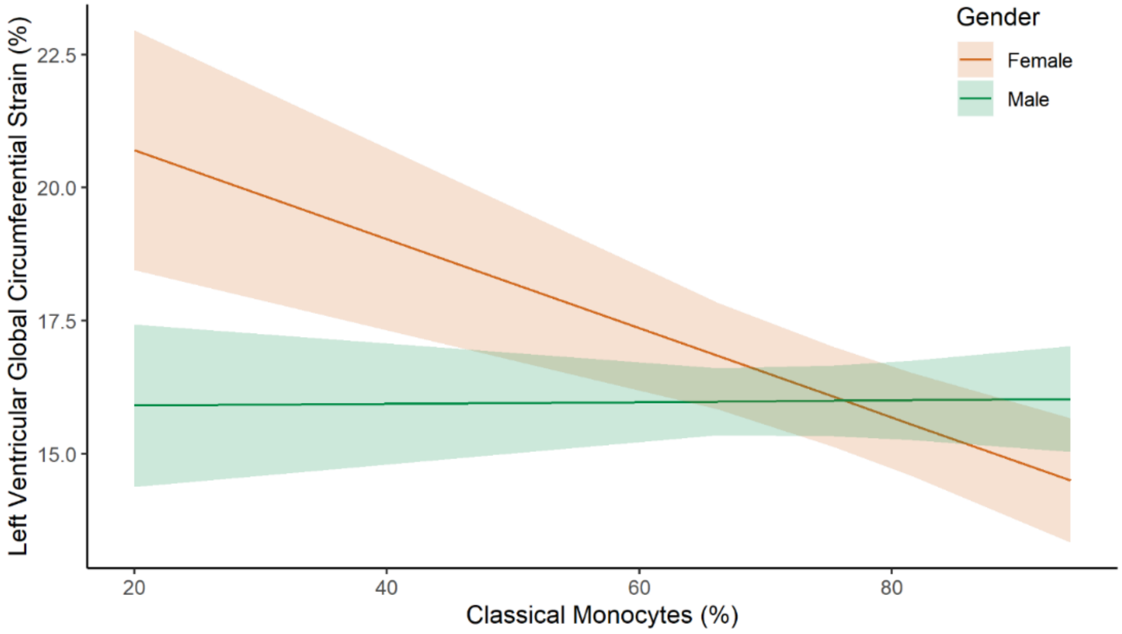

6B

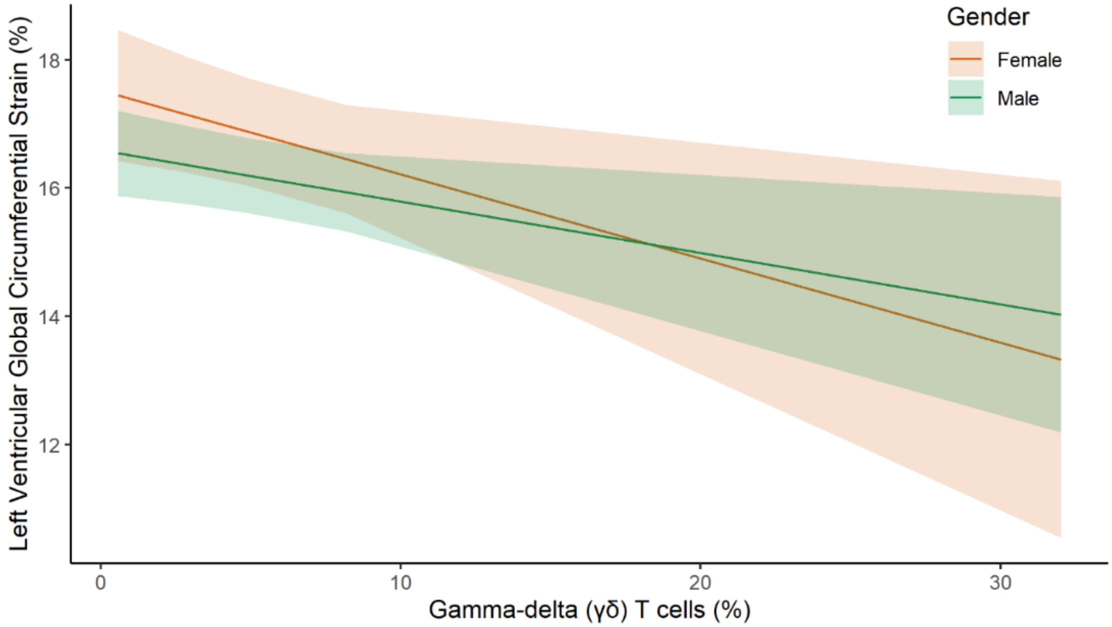

A. The association of classical monocytes with left ventricular global circumferential strain had a significant interaction with sex. There was a strong negative association between classical monocytes (%) and left ventricular global circumferential strain (%) in females and no significant association in males. B. The association of Gamma-delta (γδ) T cells with left ventricular global circumferential strain had no significant interaction with sex.

Supplemental Figure 7. Estimated Marginal Plots Evaluating the Interaction of Hypertension on the Association between Immune Cell Subsets and Global Circumferential Strain

7A.

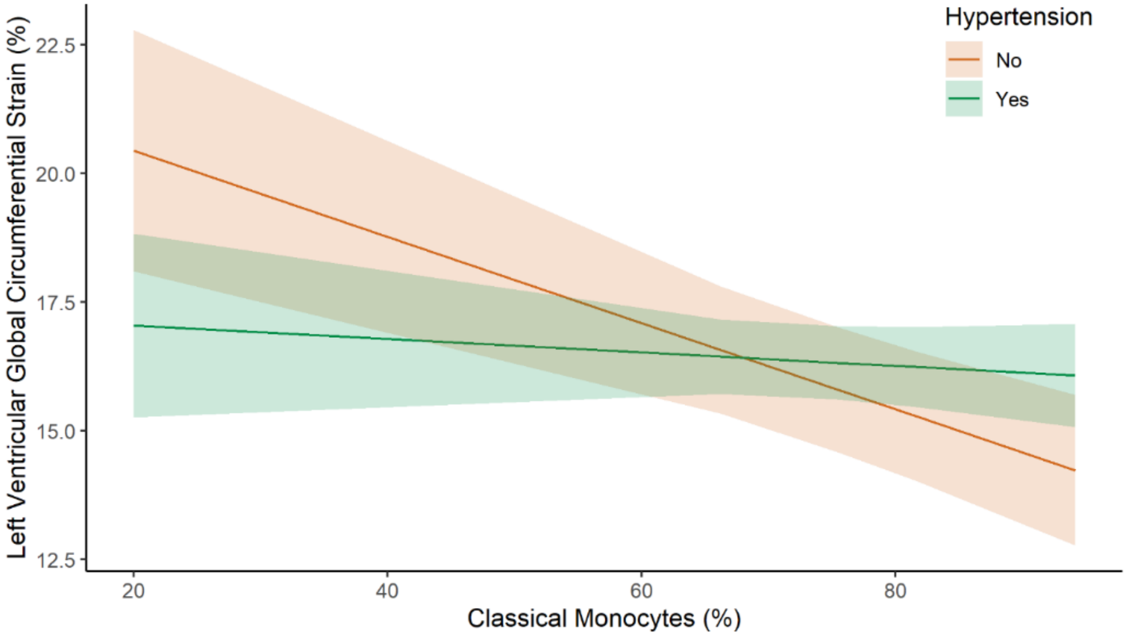

7B.

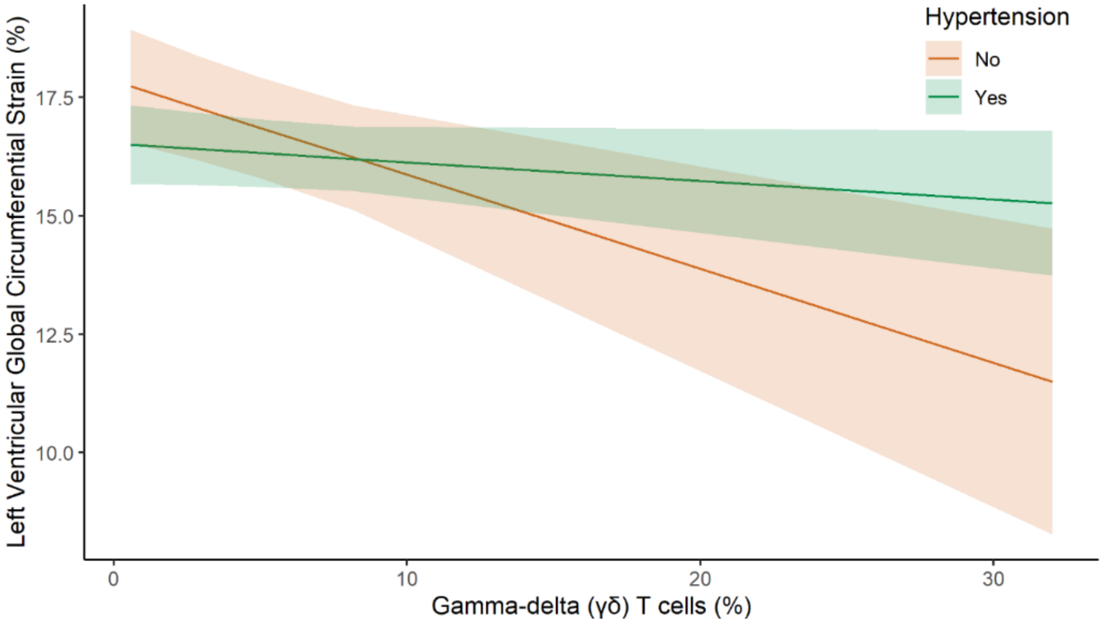

A. The association of classical monocytes with left ventricular global circumferential strain had a significant interaction with hypertension status. The was a significant association between classical monocytes (%) and left ventricular global circumferential strain (%) only in adults without hypertension. B. The association of Gamma-delta (γδ) T cells with left ventricular global circumferential strain had a significant interaction with hypertension status. There was a significant association between Gamma-delta (γδ) T cells (%) and left ventricular global circumferential strain (%) only in adults without hypertension.
